# microRNAs in axon guidance

**DOI:** 10.3389/fncel.2014.00078

**Published:** 2014-03-14

**Authors:** Archana N. Iyer, Anaïs Bellon, Marie-Laure Baudet

**Affiliations:** ^1^Center for Integrative Biology, University of TrentoTrento, Italy; ^2^Department of Physiology, Development and Neuroscience, University of CambridgeCambridge, UK

**Keywords:** miRNAs, axon guidance, axon, growth cone, neuron, development

## Abstract

Brain wiring is a highly intricate process in which trillions of neuronal connections are established. Its initial phase is particularly crucial in establishing the general framework of neuronal circuits. During this early step, differentiating neurons extend axons, which reach their target by navigating through a complex environment with extreme precision. Research in the past 20 years has unraveled a vast and complex array of chemotropic cues that guide the leading tip of axons, the growth cone, throughout its journey. Tight regulation of these cues, and of their receptors and signaling pathways, is necessary for the high degree of accuracy required during circuit formation. However, little is known about the nature of regulatory molecules or mechanisms fine-tuning axonal cue response. Here we review recent, and somewhat fragmented, research on the possibility that microRNAs (miRNAs) could be key fine-tuning regulatory molecules in axon guidance. miRNAs appear to shape long-range axon guidance, fasciculation and targeting. We also present several lines of evidence suggesting that miRNAs could have a compartmentalized and differential action at the cell soma, and within axons and growth cones.

## Introduction

Brain wiring occurs during the development of the nervous system and ensures the formation of a highly complex network of inter-communicating neurons. For these circuits to be established, neurons form remarkably accurate connections with their target cells. Initially, neurons send out cell protrusions called axons, which navigate a complex environment to reach their exact targets: a process known as axon guidance (or “pathfinding”). How do axons know where to go? Specific molecules present along the pathway act as signposts to guide axons to their final destination by either repelling or attracting the leading tip of the axon—the growth cone. These guidance cues are also capable of promoting axon fasciculation, i.e., the bundling of axons together, and interactions between axons and their substrate (Tessier-Lavigne and Goodman, 1996). Over the past two decades, genetic, biochemical and cell culture analysis have unraveled four major families of guidance molecules, which can be classified into four families: Ephrins, Semaphorins, Slits, and Netrins (Dickson, 2002). More recent works demonstrated that some morphogens, growth factors, and cell-adhesion molecules also have guidance function (Kolodkin and Tessier-Lavigne, 2011). Cue-mediated signaling leads to complex remodeling of the cytoskeleton in growth cones, which in turn regulates its directional steering and interactions with other axons, cells, and the environment (Dent et al., 2011).

The nervous system contains up to a few billions of neurons depending on the species, and each neuron is at the core of a highly complex connectome, which can receive and project to up to hundreds of thousands of synaptic partners. The startling complexity of this system has long confronted neuroscientists with the incongruity of the seemingly inadequate size of the genome of roughly 20,000 defined genes. Alternative splicing is thought to partly account for such complexity, since it can generate hundreds of isoforms from a single coding gene (Schmucker et al., 2000; Li et al., 2007). In addition to this, the non-coding regulatory regions of the transcriptome, or “dark matter” (Johnson et al., 2005), is increasingly thought to account for the complexity of the neuronal connectome at the molecular level. This includes a growing number of families of small RNAs, primarily the microRNAs (miRNAs).

miRNAs are a class of small ~22 nt non-coding RNAs that have emerged, in recent years, as key post-transcriptional regulators in most eukaryotic cells. They do so by specifically binding to mRNA through partial complementarity, thereby inhibiting transcript translation, and/or stability (Bartel, 2009). Since the discovery of the first miRNA, *lin*-4, more than 20 years ago in *C. elegans* (Lee et al., 1993; Wightman et al., 1993), hundreds of new miRNAs have been identified (Griffiths-Jones, 2004; Griffiths-Jones et al., 2008; Kozomara and Griffiths-Jones, 2011, 2013) (www.miRbase.org). Importantly, the nervous system is the site of an intricate “miRNnome,” as numerous miRNAs are enriched or specifically expressed there in time and place (Johnston and Hobert, 2003; Krichevsky et al., 2003; Chang et al., 2004b; Hsieh, 2012; Zou et al., 2013). Recent large-scale studies have further revealed that individual miRNAs fine-tune the expression of hundreds of transcripts (Baek et al., 2008; Selbach et al., 2008; Guo et al., 2010). The regulatory potential of miRNAs in developing organisms, and particularly in the nervous system, thus appears infinite. The roles of miRNAs in promoting the complexity and accuracy required for circuit formation, and axon guidance in particular, has however just started to emerge.

Here, we review a small, but compelling body of research suggesting that miRNAs are important players in axon guidance. We first examine the roles of miRNAs in key steps of axon pathfinding, namely long-range guidance, fasciculation, and targeting. We then expose some evidence which points toward the possibility that miRNAs might have a compartmentalized action in projecting neurons, in the soma, axon, or growth cone.

## Roles of miRNAs in axon guidance

### Long-range guidance

In the initial phase of axon navigation, axons must first polarize, and subsequently navigate through a complex cellular terrain containing guidance cue-expressing “guidepost” cells. Neuronal or glial cells can take on the role of guidepost cells and act as substrates or intermediary targets for the growing axon. This enables axons to extend in a directed manner rather than by passive adhesion in a step-wise manner, using mechanisms that are highly conserved in both vertebrates and invertebrates (Raper and Mason, 2010). miRNAs could impact the transcriptome of projection neurons, regulating the expression of molecules that transduce cue signaling. Alternatively, they could affect guidepost cells to regulate directly or indirectly cue expression. In this section, we review a few recent findings on different model systems suggesting multiple roles and sites for miRNA action, which regulates both the navigating neuron and its environment.

Pinter and Hindges (2010) were the first to report that miRNAs, as a class of molecules, are important for long-range axon navigation using mice retinal ganglion cells (RGCs) as a model. RGCs are the only projection neurons of the retina and convey visual information to higher brain centers. In wild type monocular species, almost all RGC axons decussate at the optic chiasm, a midline structure. Whereas in binocular species, such as mice, some axons do not cross at the chiasm, but remain ipsilateral. The midline is thus an important choice point. The authors observed that, in absence of most miRNAs, many contralateral-projecting RGC axons failed to cross at the chiasm, and instead, aberrantly navigated ipsilaterally or overshot the midline. The molecular mechanisms leading to this phenotype is unknown to date. To abolish miRNAs function, Pinter and Hindges used mutants mice where Dicer, a key enzyme responsible for the maturation of most miRNAs (Bernstein et al., 2001; Grishok et al., 2001; Ketting et al., 2001; Knight and Bass, 2001), was conditionally ablated in Rx-expressing cells including RGCs and cells forming the optic chiasm. Depletion of miRNAs in these mutants could, therefore, either lead to impaired cue expression by guidepost cells at the midline, or to altered sensitivity of RGC growth cones to midline cues following misexpression of their cognate receptors or associated signaling molecules. Several ligand-receptor pairs are known to mediate midline crossing in mice: ephrin-B2/EphB1 (Nakagawa et al., 2000; Williams et al., 2003) Slit 1/2/Robo 1/2 (Plump et al., 2002; Plachez et al., 2008) VEGF164/Neuropilin-1 (Erskine et al., 2011), Sema 6D/Nr-CAM, and Plexin A1 (Kuwajima et al., 2012). Their direct or indirect regulation by miRNAs is however unknown to date except for Neuropilin-1 (Baudet et al., 2012; Cui et al., 2012; Zhang et al., 2012) and Robo 1 and 2 (Alajez et al., 2011; Fish et al., 2011; Yang et al., 2012). Of interest, miR-218 was documented to target Slit receptors Robo 1 and 2 in non-neural cells such as cancer cells (Alajez et al., 2011; Fish et al., 2011; Yang et al., 2012) suggesting it might also play a role in neurons including axons where it is also expressed (Sasaki et al., 2013). Overall, this study is the first *in vivo* evidence to show that miRNAs may impact projecting neurons, guidepost cells, or both.

miR-9 was also recently documented to regulate the long-range guidance of thalamocortical (TCAs) and corticofugal axons (CFAs) tracts (Shibata et al., 2011). Both tracts cross the telencephalon and navigate through the internal capsule, a telencephalic structure, before reaching their final destination (Molnár et al., 2012). Migration of guidepost cells called “corridor cells” to the internal capsule is a crucial event in TCA and CFA pathfinding. These cells create a permissive corridor within the medial ganglionic eminence (MGE), a telencephalic region, normally non-permissive to the growth of TCAs, and thus enable these axons to cross the telencephalon prior to reaching their final destination (López-Bendito et al., 2006). To address the roles of miR-9 specifically in telencephalic development, Shibata, and colleagues generated miR-9-2/3 double mutant mice lacking two of the three miR-9 pre-cursors, namely miR-9-2, and miR-9-3 (Shibata et al., 2011). In miR-9-2/3 double mutants, CFAs and TCAs were severely misrouted. CFAs poorly innervated the internal capsule. Similarly, TCAs failed to reach this region, and instead aberrantly projected into the hypothalamus, an area that they normally avoid. The deregulated molecular mechanisms leading to this phenotype are unclear, and likely to be complex. Evidence suggests that the TCA and CFA aberrant projections might be attributed to impaired patterning of corridor cells, although the possibility that miR-9 acts cell-autonomously in these projecting tracts cannot be excluded. Indeed, the topographical distribution of corridor cells within the telencephalon was affected; corridor neurons were expanded or dispersed in mutant animals. In addition, corridor cell markers islet-1 and Meis2 (predicted targets of miR-9) expression appeared to be qualitatively up-regulated in miR-9-2/3 double mutant mice. The mechanistic implication of this dysregulation on the pathfinding defects observed is, however, unclear. Thus, these data suggest that miR-9 may ensure the proper development of corridor cells and in turn the accurate projection of TCA and CFA to this intermediate target. Together, this study points to the interesting possibility that long-range axon guidance defects might indirectly rise from miRNA-induced impaired patterning of guidepost cells.

Finally, *lin*-4 was recently reported to also regulate long-range guidance of the axonal projection of anterior ventral microtubule (AVM) neurons in *C. elegans* larvae (Zou et al., 2012). In wild type animals, AVM axons project to the nerve ring, a neuropil considered as the *C. elegans*' brain. Before projecting anteriorly toward their target, AVM neurons are guided by two chemotropic cues that, together, orient the axons ventrally toward the midline. SLT-1 (Slit) repels AVM axons, preventing them from projecting dorsally, and UNC-6 (Netrin) attracts AVM axons ventrally (Chang et al., 2004a). The authors examined whether *lin*-4, a miRNA expressed in AVM during axon pathfinding, is important for UNC-6-mediated axon guidance. *lin*-4 was found to inhibit UNC-6 signaling during AVM axon guidance (Zou et al., 2012). Importantly, *lin*-4 acted cell-autonomously, at least in part, and specifically in post-migrating neurons. LIN-14, a transcription factor and well-described target of *lin*-4, is also expressed in AVM neurons. LIN-14 was found to mediate *lin*-4 action on AVM guidance and to potentiate UNC-6 mediated attraction of AVM axons by acting on UNC-40 (DCC) receptors. Surprisingly, *lin*-14 did not alter *unc*-40 promoter activity. Instead, it enhanced UNC-40 protein expression via an unknown mechanism, shifting its distribution from the confined perinuclear region to the whole cell. Intriguingly, *lin*-4 and *lin*-14 are broadly expressed in *C. elegans*, and both are found in several UNC-40 guided neurons. This suggests that a *lin*-4/*lin*-14 based conserved regulatory pathway might modulate UNC-6-mediated axon attraction of other tracts. In addition, miR-125, a *lin*-4 ortholog, is also present in neurons of vertebrates (Sempere et al., 2004; Smirnova et al., 2005), indicating that this ancient microRNA may have conserved its guidance function. Overall, this study revealed that *lin*-4 regulates cue-mediated attraction by modulating the signaling pathway of a receptor to guidance cue. Importantly, it also provided evidence that miRNAs can act cell-autonomously to modulate axon guidance to the midline. In summary, a few studies have revealed that miRNAs regulate long-range axon navigation, acting cell autonomously on projecting neurons, and possibly on guidepost cells.

### Fasciculation

Pioneers axons begin their pathfinding journey in an environment devoid of axons and are the first to establish connection with the target. Follower axons arise at a later time point in development and can progress along the pathway through axon-axon contact, thereby using topographical information provided by pioneers (Pittman et al., 2008). The process by which those co-extending axons form tight bundles is called fasciculation and is thought to be mediated by various classes of molecules including neural cell adhesion molecules (NCAM) but also guidance cues (Huber et al., 2005; Luxey et al., 2013). As reviewed below, some evidence suggests that miRNAs could play a role in the formation of these fasciculated bundles.

Giraldez et al. (2005) reported that Maternal Zygotic (MZ) Dicer zebrafish mutants, devoid of maternal and embryonic sources of Dicer, exhibit several defasciculated axon tracts. Specifically, fasciculation of the post-optic commissure and hindbrain axonal scaffold, formed by longitudinal and commissural tracts, were severely disrupted in the absence of most miRNAs. Although defasciculation can lead to aberrant axonal trajectory (Huber et al., 2005), projections were correctly established at least for longitudinal hindbrain axons. In addition, early patterning and fate specification was preserved in these animals. This suggests that these defects may be linked to altered molecular programs specifically in these projecting neurons, although impaired cue expression within the axonal environment cannot be formally ruled-out. Interestingly, exogenous miR-430 family members partly rescued this phenotype. This suggests that members of this family, or other uncharacterized miRNAs, may alter the expression or signaling of molecules mediating bundling of these tracts. Such molecules may include Sema3D and its cognate receptor Neuropilin-1A, which is known to promote fasciculation of hindbrain longitudinal axons in zebrafish (Wolman et al., 2004; Kwok et al., 2012). A defasciculation phenotype of RGC axons was also observed in Rx-conditional Dicer knockout mice (Pinter and Hindges, 2010). In these animals, RGC axons failed to form a tight bundle within the retina. In addition at the midline, axons that aberrantly projected ipsilaterally were defasciculated, while axons overshooting the chiasm formed a secondary defasciculated tract. Interestingly, Sema 3D, Plexin A-1, Nr-CAM, Slit1, and 2 are implicated in the fasciculation of RGC axons (Ringstedt et al., 2000; Plump et al., 2002; Kuwajima et al., 2012) suggesting that their signaling might be derailed in Dicer mutants. Overall, miRNAs appear to regulate fasciculation, although the molecular mechanisms and the nature of the miRNAs involved are still largely elusive.

### Axon targeting

After their long journey, axons reach their final destinations. Targeting of axons to their exact partner is absolutely essential, as it ensures proper circuit formation. This process is highly complex and requires several classes of molecules that promote defasciculation and specific entry within the target region, restricts any further elongation but also prevent axons from exiting the target-area. Cue-mediated restriction of the target-area is a highly regulated process in which miRNAs have been recently shown to play a role (Baudet et al., 2012).

Using *Xenopus laevis*, Baudet et al. (2012) uncovered a miRNA based signaling pathway that regulates axon targeting of RGCs to the optic tectum. Knockdown of miR-124 neither altered the birth of RGCs nor the general progression of their differentiation. However, it appeared to affect post-mitotic RGCs axon projection. While long-range guidance was unaffected, a subset of axons failed to appropriately stall within the optic tectum. Instead, they invaded Sema3A expressing territories in the ventral border, normally repellent to these axons at this stage. The effect of miR-124 is likely to be cell-autonomous, as straying axons were observed both when miR-124 was knocked down in cells of the central nervous system (which include RGCs and tectal cells), and also when knocked down at a later developmental stage in retinal cells. In addition, growth cone responsiveness to Sema3A was impaired in miR-124 morphants. The authors also elucidated the molecular pathway mediating miR-124-regulated Sema3A repulsion. miR-124 indirectly promoted the expression of Neuropilin-1, a Sema3A receptor, at the growth cone, since its depletion decreased Neuropilin-1 levels within growth cones *in vitro* and axons *in vivo*. miR124 regulated Neuropilin-1 via the silencing of its conserved target coREST, a cofactor of the global neuronal repressor REST (RE1-silencing transcription factor). Indeed, knockdown of coREST rescued Neuropilin-1 levels at the growth cone, and also growth cone responsiveness to Sema3A, in miR-124 morphants *in vitro*. Overall, this study uncovered a complex mechanism whereby miR-124 ensures RGC axonal response to Sema3A, at the right time and place, by dynamically inhibiting coREST repression of Neuropilin-1 within maturing RGCs. It also revealed for the first time that a miRNA regulates axon guidance (targeting) *in vivo*.

### Conclusion

**Figure 1:**
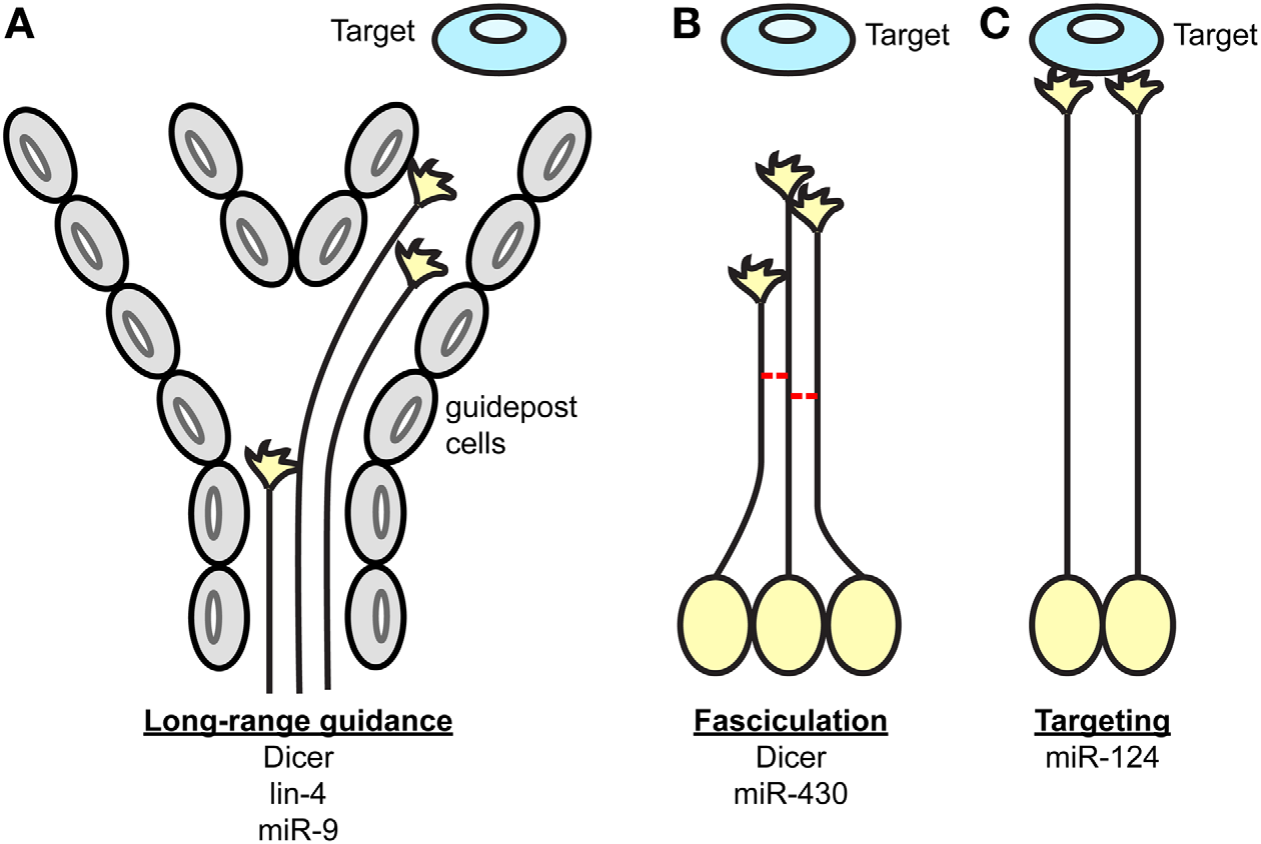
**Key processes of axon guidance regulated by miRNAs**. During axon pathfinding toward a target, miRNAs regulate **(A)** the long-range guidance of axons by acting within projection neurons and/or guidepost cells located along permissive corridors or at the midline, **(B)** the fasciculation of axons in a given tract, and **(C)** the restriction of the axonal targeting area. The components of the miRNA pathway involved in each process are specified under each scheme.

In summary, several studies have together revealed the function of miRNAs in axonal navigation to their final destinations using central nervous system projections as model (Table 1, Figure 1) (Giraldez et al., 2005; Pinter and Hindges, 2010; Shibata et al., 2011; Baudet et al., 2012; Zhang et al., 2013; Chiu et al., 2014). Earlier work took a broad approach, and knocked down the entire pool of miRNAs using a Dicer loss-of-function strategy (Giraldez et al., 2005; Pinter and Hindges, 2010). This was particularly important at that time to determine whether miRNAs, as a class of molecules, are involved in axon guidance. Although striking phenotypes were observed suggesting the importance of miRNAs in this process, the full extent of miRNAs' implication in guidance maybe somewhat underestimated for several reasons. miRNA turn-over varies, and some can be particularly stable for a long time following ablation of Dicer (Schaefer et al., 2007). In addition, recent studies have shown that miRNAs can be synthesized via a Dicer-independent mechanism (Cheloufi et al., 2010; Cifuentes et al., 2010; Yang et al., 2010)—although, only one miRNA, miR-451, is documented to employ this non-canonical pathway (Yang et al., 2010). Of interest, Dicer is also involved in small interfering (si) RNA processing from various sources such as small nuclear (sn) RNA and viral double stranded (ds) RNA (Bernstein et al., 2001; Grishok et al., 2001; Ketting et al., 2001; Knight and Bass, 2001; Li et al., 2002). Dicer loss-of-function in these initial analyses (Giraldez et al., 2005; Pinter and Hindges, 2010) could thus impair this processing also. The importance of these additional roles has yet to be demonstrated in neurons however. Later studies went on to unravel the roles of individual miRNAs in axon guidance. New insight has come from those that have explored the cell-autonomous roles of miRNAs *in vivo*; for instance directly in projecting neurons (Baudet et al., 2012; Zou et al., 2012). Future research *in vivo* should however reveal additional functions of miRNAs, and their associated mechanisms of action. In particular, it is unknown whether miRNAs modulate cue expression in the pathway, either by acting directly on post-transcriptional regulation of transcripts expressed in guidepost cells, or on their patterning. However, gaining future insight will be complicated by the fact that this field has several pitfalls. High level of redundancy of miRNA function exists, especially for those miRNAs derived from the same family (Choi et al., 2008) or the same polycistron (Ventura et al., 2008) making the identification of individual guidance miRNAs particularly difficult. Deciphering the molecular mechanisms at play represents also a hurdle, since miRNAs are often part of complex molecular networks. Overcoming these challenges will thus be crucial in the future elucidation of miRNA function in guidance.

**Table 1:** **List miRNAs and their target involved in guidance**.

**miRNA**	**mRNA**	**Age**	**Species**	**Neuron type**	Phenotype^*^	**References**
*lin*-4	LIN-14	L1 and L2 stage	*C. elegans*	AVM	Impaired long-range guidance	Zou et al., 2012
miR-124	CoREST	St 24,32,40	*X. laevis*	RGC	Impaired targeting	Baudet et al., 2012
miR-134	Xlimk1	St 22	*X. laevis*	Spinal	Loss of BDNF-induced growth cone turning	Han et al., 2011

* *upon loss of function*.

*Abbreviations: AVM, Anterior Ventral Microtubule; RGC, Retinal Ganglion Cells; st, stage; X, Xenopus*.

## Compartmentalized action of miRNAs

Numerous miRNAs appear to be differentially distributed within organisms, tissues, and cells. This is particularly true for the nervous system where miRNAs are enriched and specifically located in different regions and cell types (Krichevsky et al., 2003; Landgraf et al., 2007; Pichardo-Casas et al., 2012). Intriguingly, differential distribution is also observed at the subcellular level. Specific miRNAs are found to be enriched at synapses and dendrites compared to the cell soma (Siegel et al., 2009). This is perhaps not surprising considering that neurons are highly polarized cells with compartmentalized mRNA repertoires (Taylor et al., 2009; Zivraj et al., 2010; Gumy et al., 2011; Kaplan et al., 2013) implying that different compartments may have different regulatory requirements. Recent data have emerged suggesting that miRNAs are localized and might function within different subcellular location of projection neurons. For instance, some miRNAs may act within soma, affecting targets that have a global range of action; whilst others may have a more restricted, compartmentalized action within axons, and possibly, restricted to growth cones. The following section presents data summarizing these two possibilities.

### Somatic roles of miRNAs

Aforementioned studies have provided evidence that at least two specific miRNAs are likely to act primarily within the neuronal cell body during axon guidance. miR-124 in *Xenopus* (Baudet et al., 2012) and *lin*-4 in *C.elegans* (Zou et al., 2013) have somatic distribution within RGCs and AVM, respectively. *lin*-4 ortholog miR-125b is enriched in axons of the superior cervical ganglion (SCG) in mice (Natera-Naranjo et al., 2010) however, suggesting that the subcellular distribution might be cell or species specific. In contrast, miR-124 is enriched in the perinuclear cell soma of various neurons, compared to axons, synapses, or dendrites (Kye et al., 2007; Siegel et al., 2009; Natera-Naranjo et al., 2010), suggesting that this miRNA might have a conserved site of action. In addition, the molecular nature of the miR-124 and *lin*-4 targets strongly suggest restricted action within cell bodies, as both targets are transcription factors: coREST (Baudet et al., 2012) and *lin*-14 (Zou et al., 2012). Taken together, this suggests that miR-124 and *lin*-4 acts within neuronal cell soma of projecting neurons to regulate axonal pathfinding.

miRNAs were first described as heterochronic genes regulating the developmental timing of many *C.elegans* cell lineages (Lee et al., 1993; Wightman et al., 1993; Reinhart et al., 2000). Their roles as timers also occur in vertebrates including in neuronal lineages (Decembrini et al., 2009; Cremisi, 2013; La Torre et al., 2013). Intriguingly, miRNAs might also function as timers in in post-mitotic neurons during later developmental events (Olsson-Carter and Slack, 2010; Baudet et al., 2012; Zou et al., 2012) but also following terminal differentiation (Chiu and Chang, 2013; Zou et al., 2013). In particular, *lin*-4 and miR-124 were reported to affect the developmental aging of post-mitotic differentiating neurons during the period of axon elongation and guidance. As mentioned above, miR-124 regulates Sema3A-mediated RGC axon targeting within the tectum through transcriptional de-repression of Neuropilin-1 by coREST silencing (Baudet et al., 2012). Importantly, RGC axons gain responsiveness to Sema3A over time, as they navigate along the pathway, and this onset of responsiveness is due to the increase in Neuropilin-1 expression at the growth cone (Campbell et al., 2001). Remarkably, miR-124 may act as a timer, regulating the timetable of neuropilin-1 expression. Indeed, Baudet et al. (2012) showed series of evidence suggesting that a temporal increase of miR-124 in differentiating RGCs, during the period of guidance, accelerates the clearance of coREST transcripts, which progressively releases the transcriptional repression on Neuropilin-1. In turn, Neuropilin-1 protein levels increase at the growth cone over time. All-in-all, miR-124 indirectly determines the time at which Neuropilin-1 is expressed above a level that is necessary for growth cones to gain sensitivity to Sema3A. This mechanism enables growth cones to respond appropriately to this repellent at the right time and place.

Similarly to RGC growth cones, AVM axons progressively switch and lose responsiveness to UNC-6 toward the end of the axon guidance period (Zou et al., 2013). This loss-of-sensitivity is thought to enable axons to subsequently proceed with synaptogenesis (Zou et al., 2013). *C. elegans lin*-4 is a well acknowledged regulator of developmental timing, affecting numerous cell types (Chalfie et al., 1981; Lee et al., 1993; Wightman et al., 1993). In AVM neurons, *lin*-4, like miR-124, displays a clear dynamic temporal regulation suggesting it might also regulate developmental timing in these cells. Importantly, it starts being expressed in AVM neurons only after cell fate determination and cell migration has occurred. Moreover, the 3'UTR activity of its target, *lin*-14, is also down-regulated overtime in these cells (Zou et al., 2013). This indicates that it could act as a timer to promote neuronal differentiation and axon guidance.

Two different molecular pathways have thus been uncovered, where miRNAs appear to endorse a timer function by regulating a switch in growth cone responsiveness over time. The regulatory mechanisms leading to the dynamic expression of these two miRNAs is however unknown. It would be interesting to investigate whether a master clock, regulating this common timetable of growth cone sensitivity, exists upstream that regulate the temporal expression of these miRNAs.

### Local roles of miRNAs at the growth cone

The growth cone is a subcellular compartment that can function with a great deal of independence from the cell body, since severed growth cones can navigate on their own along the pathway for a few hours (Harris et al., 1987) and possess all the machinery necessary to respond to cues (Vitriol and Zheng, 2012). Remarkably, growth cones and axons are packed with complex and dynamically changing mRNA repertoires (Taylor et al., 2009; Zivraj et al., 2010). mRNA translation is also shown to mediate growth cone turning in response to several cues (Jung and Holt, 2011). Interestingly, mRNA regulation has emerged as an important mechanism to promote crisp growth cone steering (Jung et al., 2011). However, the identity of key molecular players, their modes of action, and the mechanisms employed by extracellular signals to modulate mRNA translation, are largely unknown. miRNAs may thus be important post-transcriptional regulators for growth cone behavior (Jung et al., 2011), since they ensure that proteins are expressed at precise levels, at the right time and place (Bartel, 2009; Ebert and Sharp, 2012). Although this has yet to be demonstrated, a few lines of evidence support this possibility.

#### miRNA profiling within axons

Recent studies have profiled miRNAs directly within developing distal axons (also comprising growth cones) using different technical approaches and biological systems (Natera-Naranjo et al., 2010; Sasaki et al., 2013; Hancock et al., 2014). These have revealed that a complex miRNome exists in distal axons and that several miRNAs are enriched (or depleted) in this compartment (Table 2). As suggested (Hancock et al., 2014), this would be consistent with the differential expression of axonal mRNA repertoires at different developmental stages or in different species (Zivraj et al., 2010; Gumy et al., 2011). High throughput profiling of miRNAs have yet to be documented. However, in these studies, several miRNAs were also detected in growth cones by fluorescent *in situ* hybridization: miR-16 and miR-221 in SCG neurons (Natera-Naranjo et al., 2010), miR-532 and miR-181a-1^*^ in E16 cortical neurons and in dissociated hippocampal neurons (Sasaki et al., 2013) and miR-132 in E13.5 DRG explants culture (Hancock et al., 2014). Importantly the list and number of enriched axonal miRNAs, in all three studies, is strikingly different. Several reasons might explain these results. First, miRNAs might be differentially distributed in axons depending on the species (rat vs. mouse), cell type (SCG, cortical, and DRG neurons) and developmental stage (P3, E16, E13.5). Second, these differences may be due to different axonal culture (compartmentalized chamber vs. neuronal ball) and profiling methodologies (microarray/qRT-PCR vs. multiplex qRT-PCR). Third, they may be due to limited coverage of the known mature miRNAs to date (miRbase release 19), and the different cut-off values used for analyses. In addition in the first two studies, the majority of miRNAs appear to be distributed in both cell body and axonal compartments, suggesting that most miRNAs might not have a preferred site of action (Natera-Naranjo et al., 2010; Sasaki et al., 2013). Intriguingly, the presence of miRNAs in axons and growth cones, and to some extent differentially expressed miRNAs derived from the same polycistron (Natera-Naranjo et al., 2010; Kaplan et al., 2013; Zhang et al., 2013), suggest that a mechanism of transport similar to that speculated for dendrites exists (Kosik, 2006). Mature miRNAs could thus be translocated along axons to growth cones either as individual molecules, as precursors, or within ribonucleoparticle bound to their targets and components of the silencing machinery. For instance, pre-miR-134 was recently documented to localize to dendrites through DEAH-box helicase DHX36-mediated transport (Bicker et al., 2013). Overall, these findings point to the possibility that miRNAs might be transported to and function within growth cones to modulate steering.

**Table 2:** **List of miRNAs enriched or depleted in axons, or present in growth cones during axon development**.

**miRNAs**	**Age**	**Species**	**Neuron type**	**Enriched/Depleted in axons^a^**	**Method used**	**References**
let-7c	P3^c^	Rat	SCG	Enriched	Microarray and qRT-PCR	Natera-Naranjo et al., 2010
let-7-e	E13.5^d^	Mouse	DRG	Enriched	qRT-PCR	Hancock et al., 2014
let-7-i	E13.5^d^	Mouse	DRG	Depleted	qRT-PCR	Hancock et al., 2014
miR-9	E16^d^	Mouse	Cortical	Depleted	Multiplex qRT-PCR	Sasaki et al., 2013
miR-9^a^	E17^d^	Mouse	Cortical	Present	qRT-PCR	Dajas-Bailador et al., 2012
miR-15b	P3^c^	Rat	SCG	Enriched	Microarray and qRT-PCR	Natera-Naranjo et al., 2010
miR-16^b^	P3^c^	Rat	SCG	Enriched	Microarray and qRT-PCR	Natera-Naranjo et al., 2010
miR-16	E13.5^d^	Mouse	DRG	Depleted	qRT-PCR	Hancock et al., 2014
miR-17	E13.5^d^	Mouse	DRG	Enriched	qRT-PCR	Hancock et al., 2014
miR-18a	E18	Rat	Cortical	Enriched	RT-PCR	Zhang et al., 2013
miR-19a	E18	Rat	Cortical	Enriched	RT-PCR	Zhang et al., 2013
miR-19b	E13.5^d^	Mouse	DRG	Enriched	qRT-PCR	Hancock et al., 2014
miR-23a	P3^c^	Rat	SCG	Enriched	Microarray and qRT-PCR	Natera-Naranjo et al., 2010
miR-23b	P3^c^	Rat	SCG	Enriched	Microarray and qRT-PCR	Natera-Naranjo et al., 2010
miR-24	P3^c^	Rat	SCG	Enriched	Microarray and qRT-PCR	Natera-Naranjo et al., 2010
miR-24	E13.5^d^	Mouse	DRG	Enriched	qRT-PCR	Hancock et al., 2014
miR-26a	P3^c^	Rat	SCG	Enriched	Microarray and qRT-PCR	Natera-Naranjo et al., 2010
miR-29a	E13.5^d^	Mouse	DRG	Enriched	qRT-PCR	Hancock et al., 2014
miR-30b	E13.5^d^	Mouse	DRG	Enriched	qRT-PCR	Hancock et al., 2014
miR-30c	E13.5^d^	Mouse	DRG	Enriched	qRT-PCR	Hancock et al., 2014
miR-34b-3p	E13.5^d^	Mouse	DRG	Depleted	qRT-PCR	Hancock et al., 2014
miR-92	E18	Rat	Cortical	Enriched	RT-PCR	Zhang et al., 2013
miR-103	P3^c^	Rat	SCG	Enriched	Microarray and qRT-PCR	Natera-Naranjo et al., 2010
miR-106a	E13.5^d^	Mouse	DRG	Enriched	qRT-PCR	Hancock et al., 2014
miR-124	P3^c^	Rat	SCG	Depleted	Microarray and qRT-PCR	Natera-Naranjo et al., 2010
miR-125a-5p	E13.5^d^	Mouse	DRG	Enriched	qRT-PCR	Hancock et al., 2014
miR- 125b	P3^c^	Rat	SCG	Enriched	Microarray and qRT-PCR	Natera-Naranjo et al., 2010
miR-127	P3^c^	Rat	SCG	Enriched	Microarray and qRT-PCR	Natera-Naranjo et al., 2010
miR-132^b^	E13.5^d^	Mouse	DRG	Enriched	qRT-PCR	Hancock et al., 2014
miR-134^a^	St22	*Xen*.	Spinal	Present	qRT-PCR, FISH	Han et al., 2011
miR-135a	E16^d^	Mouse	Cortical	Depleted	Multiplex qRT-PCR	Sasaki et al., 2013
miR-137	E16^d^	Mouse	Cortical	Depleted	Multiplex qRT-PCR	Sasaki et al., 2013
miR-138	E13.5^d^	Mouse	DRG	Enriched	qRT-PCR	Hancock et al., 2014
miR-181a-1^b^	E16^d^	Mouse	Cortical	Enriched	Multiplex qRT-PCR	Sasaki et al., 2013
miR-182	E13.5^d^	Mouse	DRG	Enriched	qRT-PCR	Hancock et al., 2014
miR-185	P3^c^	Rat	SCG	Enriched	Microarray and qRT-PCR	Natera-Naranjo et al., 2010
miR-191	E13.5^d^	Mouse	DRG	Enriched	qRT-PCR	Hancock et al., 2014
miR-195	E16^d^	Mouse	Cortical	Depleted	Multiplex qRT-PCR	Sasaki et al., 2013
miR-196c	E13.5^d^	Mouse	DRG	Depleted	qRT-PCR	Hancock et al., 2014
miR-204	P3^c^	Rat	SCG	Enriched	Microarray and qRT-PCR	Natera-Naranjo et al., 2010
miR-206	P3^c^	Rat	SCG	Depleted	Microarray and qRT-PCR	Natera-Naranjo et al., 2010
miR-218	E16^d^	Mouse	Cortical	Depleted	Multiplex qRT-PCR	Sasaki et al., 2013
miR-221^b^	P3^c^	Rat	SCG	Enriched	Microarray and qRT-PCR	Natera-Naranjo et al., 2010
miR-296	E16^d^	Mouse	Cortical	Depleted	Multiplex qRT-PCR	Sasaki et al., 2013
miR-297	P3^c^	Rat	SCG	Depleted	Microarray and qRT-PCR	Natera-Naranjo et al., 2010
miR-320	P3^c^	Rat	SCG	Enriched	Microarray and qRT-PCR	Natera-Naranjo et al., 2010
miR-328	E16^d^	Mouse	Cortical	Depleted	Multiplex qRT-PCR	Sasaki et al., 2013
miR-328	E13.5^d^	Mouse	DRG	Enriched	qRT-PCR	Hancock et al., 2014
miR-329	P3^c^	Rat	SCG	Enriched	Microarray and qRT-PCR	Natera-Naranjo et al., 2010
miR-342-3p	E13.5^d^	Mouse	DRG	Enriched	qRT-PCR	Hancock et al., 2014
miR-361	E16^d^	Mouse	Cortical	Enriched	Multiplex qRT-PCR	Sasaki et al., 2013
miR-379	E16^d^	Mouse	Cortical	Depleted	Multiplex qRT-PCR	Sasaki et al., 2013
miR-382	P3^c^	Rat	SCG	Enriched	Microarray and qRT-PCR	Natera-Naranjo et al., 2010
miR-384-5p	E13.5^d^	Mouse	DRG	Enriched	qRT-PCR	Hancock et al., 2014
miR-423	E16^d^	Mouse	Cortical	Depleted	Multiplex qRT-PCR	Sasaki et al., 2013
miR-434-3p	E16^d^	Mouse	Cortical	Depleted	Multiplex qRT-PCR	Sasaki et al., 2013
miR-434-3p	E13.5^d^	Mouse	DRG	Enriched	qRT-PCR	Hancock et al., 2014
miR-484	E13.5^d^	Mouse	DRG	Enriched	qRT-PCR	Hancock et al., 2014
miR-495	E13.5^d^	Mouse	DRG	Enriched	qRT-PCR	Hancock et al., 2014
miR-532^b^	E16^d^	Mouse	Cortical	Enriched	Multiplex qRT-PCR	Sasaki et al., 2013
miR-541	P3^c^	Rat	SCG	Enriched	Microarray and qRT-PCR	Natera-Naranjo et al., 2010
miR-680	E13.5^d^	Mouse	DRG	Enriched	qRT-PCR	Hancock et al., 2014
miR-685	E16^d^	Mouse	Cortical	Enriched	Multiplex qRT-PCR	Sasaki et al., 2013
miR-709	E16^d^	Mouse	Cortical	Enriched	Multiplex qRT-PCR	Sasaki et al., 2013
miR-720	E16^d^	Mouse	Cortical	Enriched	Multiplex qRT-PCR	Sasaki et al., 2013

a *miRNA detected (“present”) in axons and growth cones*.

b *miRNAs enriched in axons and detected in growth cones by fluorescent in situ hybridization*.

c *neuron cultured for 3–10 days in vitro*.

d *neurons cultured for 4 days in vitro*.

*Abbreviations: E, embryonic day; DRG, Dorsal Root Ganglion; SCG, Superior Cervical Ganglion; st, stage; P, postnatal day; Xen., Xenopus*.

#### miRNA RISC machinery is present in growth cones

Several studies have demonstrated the silencing machinery RISC (RNA-induced silencing complex) is present and functional in growth cone, further supporting a potential role of miRNA in growth cones. Argonautes (ago) are the catalytic components of RISC. Four Ago proteins are reported in vertebrates (mammals), each binding a similar repertoire of miRNA and mRNA targets (Meister, 2013). While ago 2 was reported to induce mRNA target cleavage with perfect complementarity with a given miRNA, the roles of ago1, 3, and 4 are still elusive. Another RISC component, GW182 protein family (TNRC6 in mammals), coordinates all downstream steps in gene silencing (Pfaff et al., 2013). Key molecules for small RNA-mediated silencing such as ago2 (Zhang et al., 2013; Hancock et al., 2014), ago 3 and 4 (Hengst et al., 2006), eIF2c (Eukaryotic Initiation Factor 2C) (Aschrafi et al., 2008) and GW182 (Dajas-Bailador et al., 2012) were detected in the embryonic and perinatal distal axons, and/or growth cones of various cell types (Table 3). In addition, one study also revealed that RISC is functional in distal axons (Hengst et al., 2006). Exogenous siRNA directed against RhoA, a small GTPase protein led to the decrease in RhoA transcript and RhoA immunoreactivity in distal axons. Importantly, FITC-labeled siRNA was not detected in proximal axons, and no RhoA mRNA knockdown was detected in the somatodendritic compartment. Taken together, these data revealed that exogenous siRNA-induced silencing exists in distal axons (Hengst et al., 2006). It would be interesting to explore whether RISC can also mediate endogenous miRNA action in this compartment, and most specifically in growth cones. Intriguingly, the RISC component Dicer is also detected in distal axons, including growth cones (Hengst et al., 2006; Zhang et al., 2013; Hancock et al., 2014). This suggests that, as in dendrites (Bicker et al., 2013), pre-miRNAs could be transported and processed into mature miRNAs, in this compartment. Axonal transfection of pre-miR-338 and pre-miR-16 indeed result in a substantial increase in their concomitant mature form in axons, suggesting that miRNA processing does occur in distal axons (Aschrafi et al., 2008; Kar et al., 2013). Several key components are thus present in growth cones and/or distal axons, and RNA interference occurs in this compartment, suggesting that miRNAs are likely to be functional there. The documented presence of RISC components Armitage, MOV10 and Dicer (Lugli et al., 2005; Ashraf et al., 2006; Banerjee et al., 2009) in pre- and post-synaptic compartments underscore that miRNAs may have broader subcellular sites of action in polarized cells like neurons.

**Table 3:** **Reports of miRNA processing machinery in neurons**.

**RISC component**	**Species**	**Neuron type**	**Age**	**References**
Dicer	Rat	DRG	E15^a^	Hengst et al., 2006
	Rat	Cortical	E18	Zhang et al., 2013
	Rat	SCG	P3^b^	Aschrafi et al., 2008
	Mouse	DRG	E13.5^b^	Hancock et al., 2014
ago2	Rat	Cortical	E18	Zhang et al., 2013
	Mouse	DRG	E13.5^b^	Hancock et al., 2014
ago3	Rat	DRG	E15^b^	Hengst et al., 2006
ago4	Rat	DRG	E15^b^	Hengst et al., 2006
GW-182	Mouse	Cortical	E17^b^	Dajas-Bailador et al., 2012

a neurons cultured for 3–7 days in vitro;

b *neurons cutlured for 3 days in vitro*.

*Abbreviations: DIV, Days in vitro; DRG, Dorsal Root Ganglion; SCG, Superior Cervical Ganglion*.

#### Do miRNAs play a local role in growth cone turning?

The presence of RISC within growth cones suggests that miRNAs could act locally within this compartment and shape the local transcriptome during axon guidance. In particular, miRNAs could regulate local translation, known to play a role in growth cone steering in response to some cues (Jung et al., 2011). Although this has yet to be clearly demonstrated, recent studies suggest that it might be the case.

miRNAs are known to regulate outgrowth in development and following injury (Wu and Murashov, 2013; Chiu et al., 2014). miRNA-mediated silencing of mRNA was recently reported to occur locally within axons to modulate outgrowth. Axonal miRNAs were initially documented to inhibit the translation of cytoskeletal regulatory molecules locally (Dajas-Bailador et al., 2012; Hancock et al., 2014). Using mice cortical neurons, Dajas-Bailador et al. (2012) first revealed that a miRNA, miR-9, modulates the translational repression of exogenous Map1b (microtubule-associated protein 1b) 3′UTR, which has a key role in the regulation of dynamic microtubules. Short BDNF stimulation modulated miR-9 expression, while inhibition of miR-9 affected axonal growth only when applied locally in axons, suggesting that BDNF affects this developmental process via local, miRNA-mediated translational control of a cytoskeletal regulator. Further support for such local mechanisms came in a recent study from Flanagan's group (Hancock et al., 2014). Hancock and colleagues reported that axon-enriched miR-132 promotes embryonic DRG axon outgrowth by targeting endogenous p120RasGAP (Rasa1), a protein involved in cytoskeletal regulation (Hancock et al., 2014). Interestingly, miR-132-induced increase in axonal Rasa1 protein level was dependent on local protein synthesis, as it was abolished in the presence of translation inhibitor applied to severed axons (Hancock et al., 2014). This demonstrated that miR-132 acts indeed within this cell compartment to regulate target translation, removing the possibility of cross-talk with the cell body. Of note, Rasa 1 was previously reported to mediate responsiveness to chemotropic cues but here, miR-132 activity did not change upon stimulation by a few guidance molecules suggesting that these findings may not be strictly transposed to the guidance field (Hancock et al., 2014). In addition, axonal miRNAs were also recently documented to promote outgrowth by silencing axonal transcripts other than cytoskeletal regulators. Using 3d rat SCG neurons, Kar and colleagues reported that axon abundant miR-16 reduces the levels of the eukaryotic translation initiation factors eIF2B2 and eIF4G2 mRNAs, specifically within axons without affecting the levels of these transcripts in the soma (Kar et al., 2013). Interestingly, axonal miR-16 reduced outgrowth, and siRNA-mediated decrease in eIF2B2 and eIF4G2 levels in axons lead to inhibition of local protein synthesis and reduced axon extension. Together, this suggests that miR-16 might regulate elongation by modulating the axonal protein synthetic system. Finally using rat E18 cortical neurons, Zhang et al. (2013) documented that axonal miR-19a, a member of the miR-17-92 cluster, regulates axon outgrowth via PTEN (phosphatase and tensin homolog), a negative regulator of the PI3K/mTOR signaling pathway. Importantly, axonal miR-19a regulates PTEN protein levels specifically within axons and not at the cell soma suggesting compartmentalized action for this miRNA. Local regulation of mRNA by miRNA has thus been reported in axons in a biological context of elongation.

The possibility that miRNA-mediated regulation of growth cone turning via local regulation of mRNA is further supported by a recent study. Several years ago, miR-134 was shown to locally modulate the size of dendritic spines of rat hippocampal cells (Schratt et al., 2006). This miRNA keeps Limk1, a kinase regulating actin polymerization, in a dormant untranslated state, and releases its repression in response to extracellular BDNF stimulation. Limk1 is thus translated, resulting in spine size increase (Schratt et al., 2006). Zheng's group recently investigated whether this mechanism is conserved in growth cones of *X. laevis* spinal neurons, where they detected this miRNA (Han et al., 2011). Similar to dendritic spines, miR-134 was found to be important for BDNF-induced growth cone attraction. In addition, miR-134 appeared to regulate protein synthesis in response to this cue, as loss- and gain-of-function of miR-134 in the whole embryo blocked protein synthesis dependent turning response of growth cones. The effect of this miRNAs on spinal neuron cell bodies cannot be formally excluded, since miR-134 was knocked down or overexpressed in whole embryos, and not exclusively in axons. Limk1, also detected in spinal growth cones, was confirmed as a *bona fide* target of miR-134 in *Xenopus* by *in vivo* luciferase assay. This suggests that Limk1 may mediate miR-134 regulation of BDNF-induced growth cone attraction. All-in-all, this study provided the first evidence, that growth cone turning can be modulated by miRNAs. It also indicated that conserved miRNA-based local control may exist in neuronal compartments, enabling the acute regulation of cytoskeletal dynamics in response to external stimuli.

Based on these recent findings, one could speculate that several possible mechanisms of mRNA regulation in growth cones exist during steering. On the one hand miRNAs could silence translation, keeping the transcript dormant until a cue is encountered, and a newly synthesized protein is asymmetrically required. Similar mechanisms of action are also reported in dendrites (Schratt et al., 2006; Siegel et al., 2009) suggesting they could be conserved across neuronal compartments. On the other end, cue-induced activation of miRNAs could lead to the inhibition of transcript translation and/or stability, when newly synthesized protein(s) are no longer required for guidance. In particular, such silencing could arrest cue-induced translation of mRNA, thereby terminating growth cone response to a given chemotropic cue. Furthermore, an asymmetric rise in local mRNA translation of a cytoskeletal protein was reported to occur at the growth cone on the side of cue exposure (Leung et al., 2006). From this, one could finally conceive that miRNAs may have an asymmetric function in this compartment, allowing silencing to occur on one side of the growth cone, and translation on the other. This putative mechanism might be unique to growth cones, as opposed to dendrites or synapses, to support directional steering.

## Conclusive remarks and perspectives

**Figure 2:**
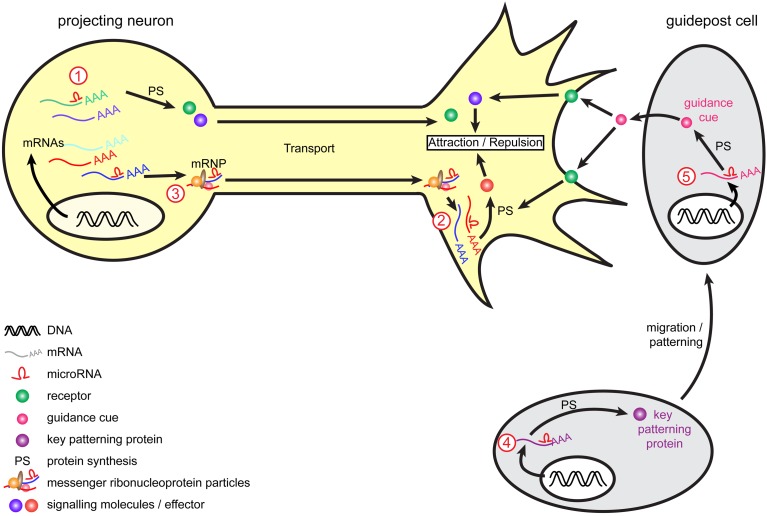
**Model of miRNA-mediated regulation of axon guidance**. During pathfinding, tight regulation of mRNAs occurs to ensure protein expression of guidance molecules at the right time and place, and enable accurate growth cone steering. Within projection neurons, transcripts are translated into the cell body and are subsequently transported within the axon to the growth cone to mediate guidance cue-induced signaling. Alternatively, mRNAs associate into messenger ribonucleoprotein particles (mRNPs) to be transported to the growth cone, where they can be locally translated. Retrograde transport of transcripts from growth cones to cell soma also exists (not represented here). miRNAs are speculated to act at multiple level. They may regulate transcripts translation and stability (1) within the cell body as suggested for miR-124 and lin-4 (Baudet et al., 2012; Zhang et al., 2013) or (2) directly within growth cones as suggested for miR-134 (Han et al., 2011) and by the presence of RISC within this compartment (Table 3). (3) As speculated (Kosik, 2006), miRNAs may translocate along the axons alone or within mRNPs (shown here) and/or be transported as pre-miRNAs and locally produced within growth cones. Guidepost cells are important partners for projection neurons, as they provide them with positional information through the expression of guidance cues. The regulation of guidepost cell transcriptome is thus of crucial importance to ensure the correct patterning of these cells and also the delivery of the right guidance cue at the right place. miRNAs could act by directly regulating the expression of guidance cues within guidepost cells (4) or by indirectly regulating molecules involved in the patterning of these cells (5), as suggested for miR-9 (Shibata et al., 2011).

In conclusion, recent studies have uncovered that miRNAs are hitherto unsuspected, important regulatory molecules in axon guidance (Figure 1) (Giraldez et al., 2005; Pinter and Hindges, 2010; Han et al., 2011; Shibata et al., 2011; Baudet et al., 2012; Zou et al., 2012). These have revealed that miRNAs are likely to have widespread and important roles, affecting different species and several projections, and when knocked out, result in varying degrees of severity in guidance errors. The studies have also shown that miRNAs are likely to regulate both guidance response to cues or cue expression. In particular, miRNAs can specifically modulate growth cone steering (Han et al., 2011; Baudet et al., 2012). To do so, they can act cell-autonomously to fine-tune the molecular make-up of projection neurons, thereby affecting their responsiveness to cues. This regulation may take place at the soma, via transcription factor regulation, which in turn, modulates expression levels of receptors to cues (Baudet et al., 2012; Zou et al., 2012). miRNAs are also suspected to act locally, and affect downstream signaling molecules of various nature including axon cytoskeleton (Han et al., 2011; Dajas-Bailador et al., 2012; Kar et al., 2013; Hancock et al., 2014). Although the evidence is more elusive, miRNAs could also modulate brain patterning, and thereby control either the presence of guidepost cells or the expression of guidance cues at key topographical locations (Pinter and Hindges, 2010; Shibata et al., 2011) (Figures 1, 2).

Guidance molecules appear to have pleiotropic roles and as such, are involved in several processes outside of the nervous system development. In particular, they are now acknowledged regulators of the immune and cardiovascular systems, including of vascular development, and angiogenesis (Adams and Eichmann, 2010; Kumanogoh and Kikutani, 2013). Guidances cues are also involved in pathological processes such as cancer and tumor progression (Chédotal, 2007; Mehlen et al., 2011). miRNAs, as key post-transcriptional regulator in most eukaryotic cells, are also implicated in these physiological and pathophysiological processes (Croce, 2009; Xiao and Rajewsky, 2009; Small and Olson, 2011) suggesting a possible mechanistic link between the two class of molecules outside of the nervous system. Importantly, several miRNAs modulate guidance cues and their receptors in cells other than neurons, including cancer cell lines but also in endothelial cells (Table 4) (Baudet et al., 2013). This raises the intriguing possibility that a given miRNA may regulate the same guidance molecules in different cellular contexts.

**Table 4:** **List of miRNAs regulating guidance molecules in non-neuronal cells**.

**miRNA**	**Target**	**Cell type**	**References**
miR-9	Neuropilin-1	Endothelial cells	Cui et al., 2012
miR 27a/b	Sema 6a	Endothelial cells	Urbich et al., 2012
miR-34	Sema 4b	Cardiomyoblast H9c2 cells	Bernardo et al., 2012
miR-181b	Neuropilin-1	Endothelial cells	Cui et al., 2012
miR-210	EphrinA3	U2OS ostesarcoma cell line	Fasanaro et al., 2008
miR-210	EphrinA3	293T cells	Pulkkinen et al., 2008
miR-214	Plexin -B1	HeLa cells	Qiang et al., 2011
miR-218	Robo1	Human breast cancer cells	Yang et al., 2012
miR-218	Robo1	Nasopharyngeal carcinoma	Alajez et al., 2011
miR-218	Robo1 and 2	HeLa cells	Fish et al., 2011
miR-218	Robo1 and 2	COS cells	Small et al., 2010
miR-218	Robo1	Human gastric cell lines	Tie et al., 2010
miR-320	Neuropilin-1	Colorectal cancer cells	Zhang et al., 2012
miR-331-3p	Neuropilin-2	Glioblastoma multiforme	Epis et al., 2009

miRNAs may have conserved important developmental roles, including axon guidance, throughout evolution. Indeed, miRNAs appear to regulate pathfinding in several species, ranging from *Drosophila* and *C. elegans* to mice and guidance miRNAs affect the same pathway in different species (e.g., the visual pathway of lower vertebrate Baudet et al., 2012 vs. higher vertebrates Pinter and Hindges, 2010). Moreover, a specific miRNA, miR-9, regulates guidance of different tracts (Shibata et al., 2011). Interestingly, two of the four miRNAs involved in guidance, miR-124, lin-4/miR-125, are highly conserved, and considered as ancient miRNAs with neural-like function (Christodoulou et al., 2010).Unsurprisingly, these miRNAs appear to have multifactorial neural action, and besides regulating guidance, also modulate earlier developmental events such as neurogenesis, cell fate determination, lineage progression, and later events such as synaptogenesis (Gao, 2010).

Guidance miRNAs appear to have a delicate regulatory action on guidance signaling pathways. The three miRNAs, for which signaling mechanisms have been uncovered, fine-tune the levels of their endogenous (Baudet et al., 2012; Zou et al., 2012) or exogenous targets (Han et al., 2011). This is very much in agreement with recent evidence that miRNAs do not act as off-switches, as originally thought from earlier studies in *C. elegans*, but rather as a rheostat, which fine-tunes protein output to functional levels (Baek et al., 2008; Selbach et al., 2008; Bartel, 2009; Guo et al., 2010). It is thus particularly interesting that mRNAs translated in the growth cone give rise to only small increases in protein levels (Jung et al., 2011), consistent with the hypothesis that miRNAs might be responsible for this. miRNAs may thus provide an additional layer of gene regulation in projection neurons, to ensure that guidance molecules are expressed at the right time and place, supporting the high level of precision critical for axon guidance.

Navigating growth cones are exposed to a myriad of cues along their pathway, and it appears that cross-talk exists between these cues and miRNAs. miRNAs can intrinsically alter the way growth cones respond to a cue, modulating the levels of their cognate receptor (Baudet et al., 2012; Zou et al., 2012). Conversely, cues also modulate miRNA's silencing potential at the growth cone. For instance, they are suspected to repress miRNA-mediated silencing, leading to local protein translation and growth cone steering (Han et al., 2011). Cues can induce a rise in miRNA levels in axons, which in turn leads to increased post-transcriptional gene silencing (Dajas-Bailador et al., 2012). The exact signaling mechanisms mediating cue-regulated miRNA action are unknown. One possibility, as has been previously shown in dendrites (Schratt et al., 2006), includes phosphorylation and activation of mTOR pathway, which is a suspected global regulator of translational activity in growth cones (Jung et al., 2012). Furthermore, cues or any external stimulus affecting the neuronal projection could also shape the miRNA repertoire of the whole neuron or specifically that of the growth cone. External stimuli were reported to either activate Dicer (Lugli et al., 2005) or degrade the RISC component (MOV10) (Banerjee et al., 2009) at the synapse- another neuronal compartment. In addition, neuronal activity was also shown to regulate miRNA turnover rate, by modulating their transcription or promoting their decay (Krol et al., 2010a), which in turn can affect dendritic remodeling (Fiore et al., 2009). A similar cue-mediated regulation of miRNA levels is conceivable in axons of projecting neurons.

Recent evidence has revealed that miRNA function could be modulated by different means. For instance, RNA-binding proteins (RNA-BP) were shown to either act in concert with miRNAs to promote silencing or, on the contrary, to compete for binding sites (Krol et al., 2010b). For instance miR-125a and Fragile X mental retardation protein (FMRP) were revealed to act cooperatively at the 3′UTR of PSD-95 mRNA to inhibit translation of this transcript within synapses (Muddashetty et al., 2011). miRNAs can also actively regulate RNA-BP in neurons (Fiore et al., 2009). RNA-BPs play important roles in developing projection neurons, ensuring mRNA transport and translational repression (Hörnberg and Holt, 2013). It is therefore conceivable that these two classes of molecules act in a coordinated manner to modulate transcript levels during axon guidance. In addition, other classes of non-coding RNAs, such as endogenous circular miRNA (Hansen et al., 2013; Memczak et al., 2013) and long-non-coding RNAs, have emerged as important regulators of miRNA action, acting as decoy or sponges that sequester, and thus buffer miRNAs in the cell (Salmena et al., 2011). Such endogenous competing RNAs (ceRNAs) might also include transcripts of protein-coding genes, whose miRNA-mediated silencing does not affect their function (Seitz, 2009; Salmena et al., 2011). In projection neurons, these ceRNAs could modulate miRNA access to their target transcript, providing an additional layer of regulation, and enabling fine-tuning of their translation. However, their existence and function in cells during axon guidance is yet to be demonstrated.

In conclusion, while the body of work reviewed here has just started to reveal the role of miRNAs in axon guidance, future research promises to unravel how these key regulatory molecules are embedded in the molecular network that enables axons to navigate to their targets with extreme precision.

### Conflict of interest statement

The authors declare that the research was conducted in the absence of any commercial or financial relationships that could be construed as a potential conflict of interest.
